# Ultrasmall superparamagnetic iron oxide nanoparticles for enhanced tumor penetration†

**DOI:** 10.1039/d2tb02630a

**Published:** 2023-03-03

**Authors:** Xue Feng, Yuxiang Xue, Sevil Gonca, Kunlang Ji, Mei Zhang, Francisco R. García-García, Quan Li, Yi Huang, Konstantin V. Kamenev, Xianfeng Chen

**Affiliations:** a School of Engineering, Institute for Bioengineering, University of Edinburgh, The King's Buildings Edinburgh EH9 3JL UK Michael.Chen@ed.ac.uk; b School of Chemistry, Centre for Science at Extreme Conditions, University of Edinburgh, The King's Buildings Edinburgh EH9 3FD UK; c School of Engineering, Institute for Materials and Processes, University of Edinburgh, The King's Buildings Edinburgh EH9 3JL UK; d School of Engineering, Institute for Energy Systems, University of Edinburgh, The King's Buildings Edinburgh EH9 3JL UK; e School of Engineering, Centre for Science at Extreme Conditions, University of Edinburgh, The King's Buildings Edinburgh EH9 3FD UK

## Abstract

The intrinsic pathological characteristics of tumor microenvironments restrict the deep penetration of nanomedicines by passive diffusion. Magnetophoresis is a promising strategy to improve the tumor penetration of nanomedicines aided by the external magnetic propulsive force. However, the research thus far has been focused on large nanoparticles, while ultrasmall superparamagnetic iron oxide (Fe_3_O_4_) nanoparticles (<∼20 nm) exhibit better performance in many applications such as cancer diagnosis and treatment. Herein, we aim to determine and understand the penetration of ultrasmall Fe_3_O_4_ nanoparticles with various sizes, shapes, surface charges and magnetizations in a 3D tumor spheroid model. The behaviour of the nanoparticles of three sizes (10, 15 and 21 nm), two shapes (spherical and octahedral), and opposite surface charges (negative and positive) was investigated. The results demonstrate that magnetically directed penetration works effectively on ultrasmall Fe_3_O_4_ nanoparticles. In the absence of a magnetic field, the shape and the surface charge of the ultrasmall magnetic nanoparticles have a more pronounced effect on their penetration compared to their dimensions. While in the presence of a magnetic field, the advantage of larger magnetic nanoparticles was obvious because they experience higher magnetic driving force due to their higher magnetic moments. Overall, relatively large (21 nm), spherical, and positively charged ultrasmall Fe_3_O_4_ nanoparticles showed greater penetration in tumors under a magnetic field. Furthermore, our findings suggest that the penetration efficiency of Fe_3_O_4_ nanoparticles is closely related to their cellular internalization ability. Therefore, optimization of the cellular uptake and of the magnetization of magnetic nanoparticles should be considered simultaneously for maximizing their penetration in tumor tissue through magnetophoresis.

## Abstract 2

This work suggests that, in the presence of a magnetic field, the size and magnetization of ultrasmall superparamagnetic Fe_3_O_4_ nanoparticles play dominant roles in tumor penetration.

## Graphical abstract

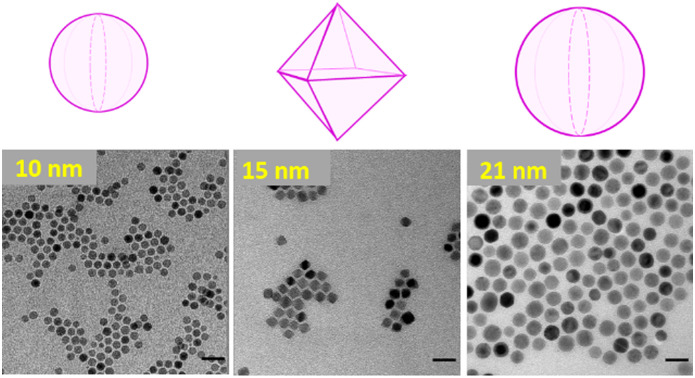

## 1. Introduction

Over the past few decades, nanomedicines with multiple functionalities have brought about impressive benefits to both cancer diagnosis and treatment. However, limited intratumoral penetration due to the complex tumor extracellular matrix and high interstitial fluid pressure severely affects the therapeutic efficacy of nanomedicines in cancer treatment. As we highlighted in a previously published review, the average penetration depth of nanomedicines in a mice xenograft tumor model is only around 130 μm measured from tumor vessels (without external force assistance).^1^ Therefore, improving the intratumoral penetration of nanomedicines and enabling them to achieve maximum therapeutic efficacy are urgent and tough tasks in cancer research.

To improve the penetration of nanomedicines in tumor tissue, much effort has been made by previous researchers by tailoring the physicochemical properties of nanoparticles. For example, many studies have proved that nanomedicines with smaller sizes and positive surface charge are associated with better penetration in tumor tissue.^2–4^ Beyond tuning the physicochemical properties of nanomaterials, magnetic nanoparticles gained extensive interest because of their unique function of autonomous movement under the control of an external magnetic field.^5–8^ Therefore, various magnetic nanorobots/motors/swimmers have been developed to perform different motion patterns under different magnetic field designs.^9–11^ Beyond passive diffusion, an external magnetic field can exert an efficient driving force on magnetic nanomedicines to promote their penetration into tumor tissue, and this process also possesses advantages of controllability and programmability.^12–18^

Iron oxide (Fe_3_O_4_) nanoparticles are the most commonly used magnetic nanoparticles in biomedicine, and several compositions have been approved for clinical use for magnetic resonance imaging (MRI), iron deficiency treatment, and thermotherapy.^19–21^ Fe_3_O_4_ nanoparticles can be mainly categorized as orally large (∼300 nm to 3.5 μm), standard (∼40 nm to 150 nm), and ultrasmall (∼<40 nm) nanoparticles.^22–24^ The standard and ultrasmall nanoparticles are the most frequently used Fe_3_O_4_ nanoparticles and are optimal for intravenous administration, whereas particle sizes below 10 nm or above 200 nm are readily removed by renal clearance or detained by the spleen.^25^ When the size is approximately below 20 nm, iron oxide nanoparticles display superparamagnetism.^26^ With regard to *in vivo* aspects, superparamagnetic Fe_3_O_4_ nanoparticles exhibit several advantages: (i) they lose their magnetization when the external magnetic field is removed, which reduces their aggregation tendency *in vivo* due to magnetic attraction, (ii) they possess high effective surface area for drug loading, (iii) their enhanced magnetic sensitivity enhances the signal in MRI, and (iv) their ultrasmall size leads to good diffusion in intercellular space.^27,28^ However, for magnetic field gradient assisted penetration, the size of the magnetic nanoparticle is a trade-off influence factor. In theory, a smaller size is good for tumor interstitial transport, but the smaller size also results in lower magnetization.^29^ The magnetic force acting on a magnetic nanoparticle can be defined using eqn (1):1
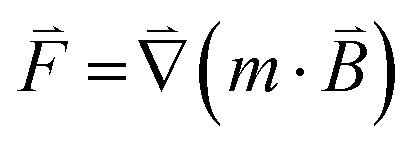
Each magnetic nanoparticle can be considered as an “equivalent” point dipole, *m*,^30^ which equals to:

2 *m* = *V*_NP_ × *M*_NP_

If it is assumed that the magnetic field (*B⃑*) setup is the same, the magnetic force on the nanoparticles is relative to their size and shape (*i.e.*, volume, *V*_NP_) as well as magnetization (*M*_NP_).

Previous works have reported that the magnetically actuated penetration of magnetic Fe_3_O_4_ nanoparticles is size-dependent, but their investigated size ranges were only limited to large and standard Fe_3_O_4_ nanoparticles.^31,32^ An investigation of ultrasmall Fe_3_O_4_ nanoparticles has so far been overlooked despite their greater performance in drug delivery compared to large nanoparticles.

Hence, in this work, we explored the penetration behaviour of ultrasmall magnetic nanoparticles under a magnetic field. We prepared three sizes of ultrasmall Fe_3_O_4_ nanoparticles comprising two shapes and two different surface charges and systematically studied the entangled relationships across different sizes, morphologies, surface charges and magnetizations of these ultrasmall superparamagnetic Fe_3_O_4_ nanoparticles during their penetration in tumor tissue under an applied magnetic field.

## 2. Materials and methods

### 2.1. Materials

Iron(iii) chloride hexahydrate (FeCl_3_·6H_2_O), iron(iii) acetylacetonate (Fe(acac)_3_), oleic acid (OA, 90%), 1-octadecene, docosane, sodium oleate, hexane, benzyl ether, 2,3-dimercaptosuccinic acid (DMSA), polyethylenimine (branched), trimethylamine, 1-ethyl-3-(3-dimethylaminopropyl)carbodiimide (EDC), *N*-hydroxysuccinimide (NHS), potassium ferrocyanide, cystamine dihydrochloride and rhodamine B isothiocyanate were purchased from Sigma-Aldrich. Nuclear fast red was purchased from ABCAM. All reagents were used without further purification. Distilled water was used for all experiments.

### 2.2. Preparation of oleic acid-coated superparamagnetic iron oxide (Fe_3_O_4_–OA) nanoparticles of different sizes and shapes

**Scheme 1:**
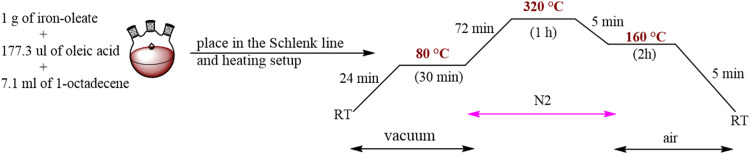
The synthesis and heating procedures of 10 nm Fe_3_O_4_–OA nanoparticles.

Spherical Fe_3_O_4_–OA nanoparticles of two different sizes (10 nm and 21 nm) were synthesized by a previously used thermal-decomposition method.^33^ The preparation method included two steps. First, the iron-oleate complex was synthesized following the previously described protocol.^33^ Second, to obtain Fe_3_O_4_–OA nanoparticles, 1 g of the above synthesized iron-oleate complex and 177.3 μL of oleic acid were added to 7.1 mL of 1-octadecene (for the preparation of 10 nm nanoparticles) or docosane (for the preparation of 21 nm nanoparticles). The mixture was first heated to a lower temperature (80 °C) and kept for 30 min to melt the solvent under vacuum conditions. Then the mixture was further heated to 320 °C (10 nm) or 380 °C (21 nm) with a heating rate of 3 °C min^−1^ and kept refluxing for 1 h (10 nm) or 5 min (21 nm) under a N_2_ environment. Then the solution was rapidly cooled down to 160 °C and kept for another 2 h before cooling the solution to room temperature. As an example, the detailed heating procedures for synthesis of 10 nm nanoparticles are illustrated in Scheme 1. The heating and cooling processes were achieved using a temperature process controller, ESM-4450 (EMKO Elektronik A.Ş., Turkey). The nanoparticles were then collected by adding an excess of ethanol followed by washing three times in a solution of 1 : 3 hexane–ethanol. After washing, the resulting nanoparticles were dissolved in hexane and stored at 4 °C.

Nonspherical Fe_3_O_4_–OA (octahedral shape) nanoparticles were synthesized following a previously described protocol.^34^ First, 1.88 g of Fe(acac)_3_ was dissolved in 17 mL of benzyl ether followed by addition of 4.38 mL of oleic acid. Second, the mixture was heated to 165 °C and kept for 30 min in a nitrogen environment. Third, the mixture was further heated to 280 °C followed by refluxing for 30 min. Fourth, the solution was cooled down to room temperature, and the obtained nanoparticles were washed with hexane/ethanol mixed solution three times. The nanoparticles larger than 20 nm were precipitated and removed by centrifugation after adding toluene because of their ferromagnetic properties (aggregation). Finally, the nanoparticles with size smaller than 20 nm were isolated and collected for further use.

### 2.3. Surface modification of magnetic nanoparticles

Two surfactants, *meso*-2,3-dimercaptosuccinic acid (DMSA) and polyethylenimine (PEI, *M*_n_ = 800), were used to replace oleic acid from the surface of the Fe_3_O_4_ nanoparticle to produce water-soluble Fe_3_O_4_ nanoparticles with negative and positive surface charges, respectively.

To prepare Fe_3_O_4_–DMSA nanoparticles, 10 mg of DMSA was dissolved in 2 mL of acetone and mixed with 10 mg of Fe_3_O_4_–OA nanoparticles in 3 ml of hexane in a glass vial. Five μL of trimethylamine was added to the above mixture and sonicated at 50 °C for 40 min. After the sonication process, a black precipitate (Fe_3_O_4_–DMSA nanoparticles) was observed and collected with a magnet. The Fe_3_O_4_–DMSA nanoparticles were washed one time with acetone and with distilled water twice. The nanoparticles were collected by centrifugation with 7200 rcf for 30 min. Then the Fe_3_O_4_–DMSA nanoparticles were re-dispersed in water, and the pH was adjusted to 10 with 1 M NaOH to deprotonate the carboxylic groups of DMSA. The nanoparticle solution became transparent at pH 10. Then, the pH was decreased to 7 with 1 M HCL for long-term storage at 4 °C.

To prepare Fe_3_O_4_–PEI nanoparticles, 10 mg of Fe_3_O_4_–OA nanoparticles and 100 mg of branched PEI with a molecular weight of 800 (PEI_800_) were mixed in 20 mL of chloroform, and the mixture was stirred at room temperature for 48 h to carry out ligand exchange reaction. After the reaction, the Fe_3_O_4_–PEI nanoparticles were collected by adding hexane and centrifugation. The precipitated nanoparticles were dried under vacuum and dispersed in water. The unbound PEI polymers were removed by ultrafiltration with a membrane of 10 kDa molecular cut-off weight (Amicon® Ultra; Merck Millipore Ltd).

### 2.4. Characterization of synthesized nanoparticles

The morphology and size of synthesized nanoparticles (Fe_3_O_4_–OA, Fe_3_O_4_–DMSA, and Fe_3_O_4_–PEI) were characterized by transmission electron microscopy (TEM, JEOL TEM-1400). To prepare TEM samples, nanoparticle solution was dropped onto a copper grid with a carbon film and air-dried at room temperature. The particle size was measured using ImageJ software (*n* > 30), and the data obtained were analyzed as the mean ± standard deviation.

The zeta potential was determined using a Malvern dynamic laser scattering instrument (DLS, Zetasizer Nano ZS 90, Malvern, UK). Samples were taken in folded capillary zeta cell cuvettes (Malvern, DTS1070) and analyzed at 25 °C (*n* = 3) in deionized water.

The magnetic properties of Fe_3_O_4_ nanoparticles were measured with a vibrating sample magnetometer (VSM, Quantum Design PPMS® DynaCool™ measurement system). The magnetization curves were recorded at 37 °C (310 K), with an applied magnetic field of up to 3 T.

### 2.5. Determination of the iron concentration of Fe_3_O_4_ nanoparticles

To determine the iron concentration of Fe_3_O_4_–DMSA and Fe_3_O_4_–PEI nanoparticles in suspensions, a spectrophotometric method based on the Prussian blue reaction was used. 40 μL of Fe_3_O_4_ nanoparticle solution was mixed with 460 μL of 6 M HCL and kept at 37 °C for 24 h to solubilize iron in the nanoparticles and oxidize Fe^2+^ to Fe^3+^. Then, 100 μL of the mixture was mixed with 100 μL of 5% potassium ferrocyanide. After 15 min of incubation, the optical density (O.D.) at 630 nm was read using a plate reader (CLARIOstar® plus). A standard curve of iron in 6 M HCL was produced by using iron chloride (FeCl_3_·6H_2_O) as the standard solution. Solutions with different Fe concentrations were prepared and mixed with an equal volume of 5% potassium ferrocyanide for 15 min. The O.D. at 630 nm was measured and plotted as a function of Fe concentration (Fig. S1, ESI†).

### 2.6. Preparation of fluorescence labelled nanoparticles

**Scheme 2:**
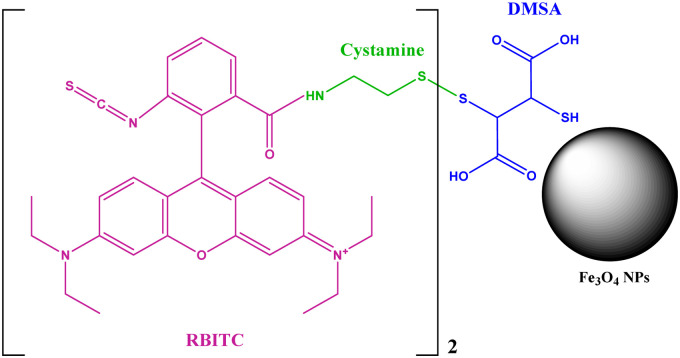
Illustration of labelling Fe_3_O_4_–DMSA nanoparticles with RBITC.

A fluorescent dye, rhodamine B isothiocyanate (RBITC), was used to label Fe_3_O_4_–DMSA nanoparticles for fluorescence imaging. To label Fe_3_O_4_–DMSA nanoparticles, cystamine was selected as a linker to connect RBITC and Fe_3_O_4_–DMSA nanoparticles. RBITC was first conjugated with cystamine through amide bond formation. The thiol groups on the DMSA are available for coupling with cystamine–RBITC *via* the formation of a disulphide bridge. The procedure is displayed in Scheme 2. Briefly, 4 mg of RBITC was dissolved in 6 ml of water, and then 1.65 mg of EDC and 3.8 mg of cystamine dihydrochloride were added. The pH of the mixture was adjusted to 7 followed by reaction for 3 h under stirring. Then, 12 mg of Fe_3_O_4_–DMSA nanoparticles was added to the mixture and reacted for an extra 2 h at room temperature. Finally, the Fe_3_O_4_–DMSA–RBITC nanoparticles were collected with a magnet and washed with water until the supernatant became transparent.

### 2.7. Cell culture

A MCF-7 (human breast adenocarcinoma cancer) cell line was bought from ATCC®. MCF-7 cells were maintained in DMEM medium containing 10% FBS, 1% penicillin G and 1% streptomycin and cultured at 37 °C under a humidified 5% CO_2_ atmosphere.

### 2.8. Cytotoxicity assay

The cytotoxicity of different Fe_3_O_4_ nanoparticles was assessed by analyzing the MTT cell viability. Briefly, 1 × 10^4^ per well MCF-7 cells were seeded into 96-well plates containing 200 μL of growth medium and incubated for 24 h at 37 °C/5% CO_2_. Then, the old medium in each well was replaced with 150 μL of fresh medium (without FBS) containing nanoparticles with different Fe concentrations of 40, 80, and 160 μg mL^−1^. After incubating for 24 or 48 h, 15 μL of MTT (5 mg mL^−1^) was added to each well and incubated for 4 h at 37 °C. Subsequently, the medium in each well was aspirated, and 150 μL of DMSO was added to dissolve the formed formazan. Finally, the plate was read at 570 nm (O.D.) using a plate reader and the cell viability was calculated. The data are expressed with the mean values of five samples.

### 2.9. Investigation of cellular uptake of Fe_3_O_4_ nanoparticles *via* Prussian blue staining

For time-dependent cellular uptake studies, MCF-7 cells in growth medium (100 μL) were seeded on 18 × 18 mm coverslips, which were placed in a 6-well plate with a density of 2 × 10^4^ cells per coverslip and incubated at 37 °C in a humidified 5% CO_2_ atmosphere for 20 min to allow cell adhesion. 1.5 mL of DMEM was then added to each well for further 24 h incubation. After removing the medium, the cells were incubated with 20 μg Fe per mL of different Fe_3_O_4_–DMSA and Fe_3_O_4_–PEI nanoparticles for 1, 3, 5, and 7 h. The cells were washed twice with PBS and fixed with 4% paraformaldehyde for 20 min, and then washed. Next, the Fe_3_O_4_ nanoparticles in cells were stained with Prussian blue. Briefly, the cells were incubated with a freshly prepared mixture of 2% potassium ferrocyanide and 2% HCL (1 : 1 volume mix) for 30 min at room temperature. After washing three times, the cells were counterstained with 0.1% nuclear fast red for 10 min. Then, the coverslips were taken out from the 6-well plate, dehydrated in 95% and 100% ethanol (twice for each, 1 min), cleared in xylene (three times, 1 min), and mounted in permount mounting medium (Fisher Chemical™ SP15-100). The Prussian blue stained cell samples were observed using a QImaging Micropublisher 3.3 colour camera installed on a microscope (Zeiss Axio Imager, Germany).

The relative area of the stained Fe_3_O_4_ nanoparticles in each cell was quantified using ImageJ using the blue stained area divided by the total cell area (determined by the nuclear fast red staining). Thirty cells were calculated for each Fe_3_O_4_ nanoparticle group, and the average was obtained.

### 2.10. Quantitative analysis of iron uptake in cells by the colorimetric titration method

In order to quantitatively analyze the uptake volume of Fe in cells, a colorimetric titration method was used, which is also based on Prussian blue reaction. 3 × 10^5^ per well MCF-7 cells in the DMEM medium (1.5 mL) were seeded on a 6 well plate and incubated at 37 °C/5% CO_2_. After 24 h of incubation, the cells were treated with different Fe_3_O_4_ nanoparticles with an iron concentration of 60 μg mL^−1^ for 1, 3, 5, and 7 h. The cells were collected with trypsin, washed twice with PBS, and counted with a hemocytometer. Then, the cells and intracellular nanoparticles were digested with 200 μL of 6 M HCL at 37 °C for 48 h followed by at 80 °C for 4 h (vortex and sonication were used to homogenize the cell mixture). After that, the mixture was allowed to sit for 30 min, and then 100 μL of the top solution was transferred to a 96 well plate, and 100 μL of 5% potassium ferrocyanide was added. After 15 min of incubation, the absorbance at 630 nm was measured and compared with the standard curve to calculate the iron concentration. The iron uptake results were normalized and expressed as μg per 10^5^ cells.

### 2.11. Development of a 3D tumor spheroid model

The *in vitro* penetration of different nanoparticles was performed using a tumor spheroid model which was produced by a hanging drop method. 30 μL of 3–10 × 10^5^ MCF-7 cells in DMEM containing 20% FBS were seeded on the inverted lid of a 65 mm Petri dish to form a drop, and 20 drops were produced per dish. The Petri dish was filled with PBS to prevent the evaporation of the hanging drops. The lid was then inverted onto the dish and incubated at 37 °C/5% CO_2_ for 4–6 days until spheroids had formed (confirmed by optical microscopy).

### 2.12. The penetration of different sized and shaped Fe_3_O_4_–DMSA nanoparticles in tumor spheroids with or without a magnetic field

The RBITC-labelled Fe_3_O_4_–DMSA nanoparticles were used to study the penetration of different sized and shaped Fe_3_O_4_ nanoparticles under the effect of a magnetic field. The three types of Fe_3_O_4_–DMSA–RBITC nanoparticle solutions prepared in 200 μL of DMEM medium (50 μg mL^−1^) without FBS were added to a 96 well plate. The tumor spheroids were transferred from the Petri dish to the 96 well plate containing nanoparticle solutions. To investigate the influence of magnetic field on the penetration efficacy of different types of Fe_3_O_4_ nanoparticles, a magnet (210–240 mT, measured using a magnetometer) was used to supply a magnetic field gradient. After 8 hours (or 16 hours) of incubation with nanoparticles, the magnet was placed under the bottom of the 96 well plate where spheroids were located and incubated for another 16 hours (or 8 hours) so that the total penetration time was 24 h in both cases. The spheroids in the control groups were incubated with the same types of Fe_3_O_4_ nanoparticles for 24 h without the magnetic field. The spheroids were then washed with PBS twice, fixed with 4% paraformaldehyde for 40 min, and transferred to a 24-well glass-bottom confocal plate before being observed by confocal laser scanning microscopy (ZEISS, 880, LSM, Germany). The Z-stack function was used to scan the Fe_3_O_4_–DMSA–RBITC nanoparticles in the whole 3D spheroid model layer by layer with a 5 μm scanning interval (10× objective). The images were analyzed using ImageJ. The fluorescence intensity of Fe_3_O_4_–DMSA–RBITC nanoparticles in the central area of each spheroid section (a fixed circular area with a diameter of 400 μm drawn in ImageJ) was quantified using ImageJ.

### 2.13. The penetration of different surface charged Fe_3_O_4_ nanoparticles in tumor spheroids with or without a magnetic field

Due to the fluorescent dye loading efficiencies for Fe_3_O_4_–DMSA and Fe_3_O_4_–PEI nanoparticles being different, it would have been inaccurate to compare the penetrations by measuring the fluorescence intensity. Therefore, the Prussian blue staining method was used to compare the penetration of DMSA and PEI coated nanoparticles in spheroids by slicing spheroids into sections. Spheroids were first incubated with Fe_3_O_4_–DMSA and Fe_3_O_4_–PEI nanoparticles DMEM solution (Fe concentration = 90 μg mL^−1^) for 8 h, followed by 16 h of further incubation with or without a magnetic field. The spheroids were washed with PBS and fixed with 4% paraformaldehyde for 1 h. To prepare the histological sections of spheroids, the spheroids were embedded in 1% agarose and paraffinized, and 5 μm thick sections were sliced. Then, the sections were dewaxed and rehydrated in xylene (2 × 10 min), a series of ethanol solutions (100%, 95%, 90%, 80% and 70%, 5 min for each), and deionized water (5 min). The Fe_3_O_4_ nanoparticles in spheroid sections were stained with a mixture of 10% potassium ferrocyanide and 20% HCl (1 : 1 volume mix) for 30 min and washed twice with water. The cytoplasm was counterstained with 0.1% nuclear fast red for 10 min and rinsed with water. All slides were mounted in Permount™ mounting medium with coverslips and observed using a QImaging Micropublisher 3.3 colour camera installed on a microscope (Zeiss Axio Imager, Germany).

The relative area of the stained Fe_3_O_4_ nanoparticles in a certain depth of spheroids (20 μm and 100 μm) was quantified using ImageJ using the blue stained area divided by the central area of the spheroid section (a fixed circular area with a diameter of 600 μm drawn using ImageJ).

### 2.14. Statistical analysis

Statistical analysis between groups was conducted using the Student's *t*-test for paired groups and one-way analysis of variance (ANOVA) for multiple groups using Excel version 2016. *P*-values less than 0.05 were considered statistically significant.

## 3. Results and discussion

### 3.1. The properties of six types of paramagnetic Fe_3_O_4_ nanoparticles

**Fig. 1:**
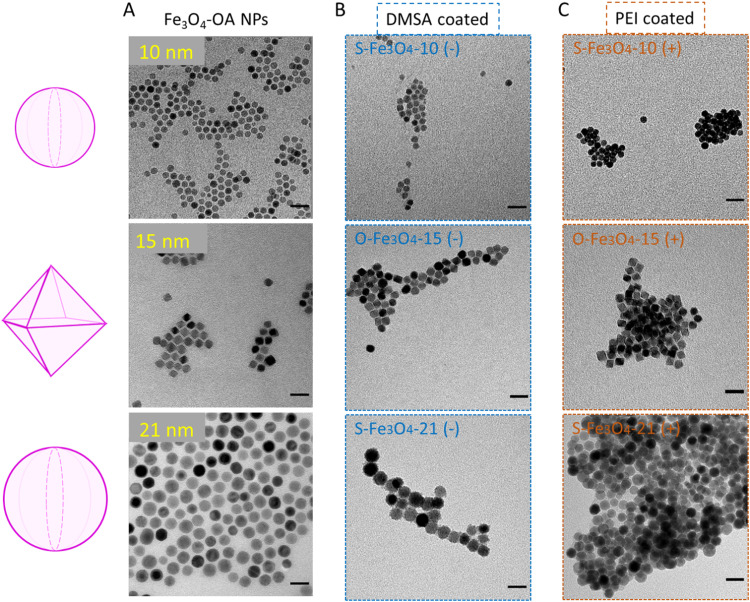
The schematic representation and TEM images of spherical 10 nm, octahedral 15 nm, and spherical 21 nm Fe_3_O_4_ nanoparticles. (A) Oleic-acid coated nanoparticles. (B) DMSA coated nanoparticles. (C) PEI coated nanoparticles. The scale bar indicates 30 nm.

**Fig. 2:**
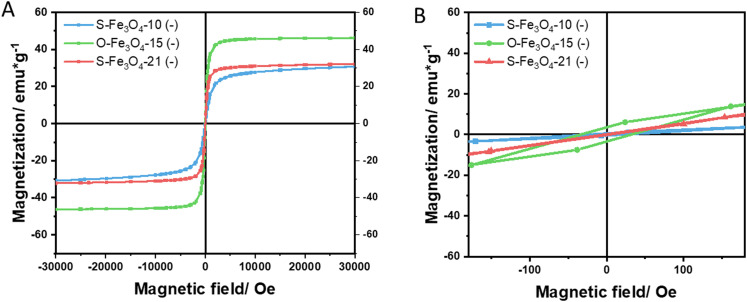
The magnetization curves of (A) three types of Fe_3_O_4_–DMSA nanoparticles at 310 K. (B) A zoomed-in graph of the low magnetic field region.

The decomposition speed of the metal precursor and the competitive growth speeds of [100] and [111] facets determine the final size and shape of the Fe_3_O_4_ nanocrystal, respectively.^35^ In this study, oleic acid-coated Fe_3_O_4_ (Fe_3_O_4_–OA) nanoparticles of three sizes and two shapes were synthesized. The average sizes and shapes of the nanoparticles were 10 ± 1 nm (spherical), 15 ± 1.2 nm (octahedral) and 21 ± 2.9 nm (spherical) determined by transmission electron microscopy (TEM) images (Fig. 1A and Fig. S2, ESI†). For the octahedral nanoparticles, the particle size was defined as the length between two opposite vertices (the edge length was 12 nm). The corresponding average volumes of the three types of nanoparticles were approximately 524 (spherical 10 nm), 815 (octahedral 15 nm) and 4849 (spherical 21 nm) nm^3^. The three types of nanoparticles were then surface modified with DMSA and PEI_800_ to obtain negative (denoted S-Fe_3_O_4_-10 (−), O-Fe_3_O_4_-15 (−), and S-Fe_3_O_4_-21 (−)) and positive (denoted S-Fe_3_O_4_-10 (+), O-Fe_3_O_4_-15 (+), and S-Fe_3_O_4_-21 (+)) surface charges. The TEM images (Fig. 1B and C) showed that all the nanoparticles maintained their size and shapes after surface modification. Table S1 (ESI†) lists the hydrodynamic sizes and zeta potentials of all nanoparticles determined by dynamic light scattering (DLS). The hydrodynamic size refers to the size of nanoparticles diffusing in solution which is larger than the size determined by TEM because of the hydration layer covering the surface of nanoparticles. For all three types of positively charged nanoparticles, the hydrodynamic sizes showed a large increase compared to TEM sizes. The zeta potential of the negatively and positively charged nanoparticles ranged from −30.8 to −49 mV and 22.7 to 30.5 mV, respectively. The magnetic properties of three types of nanoparticles (represented by DMSA-coated Fe_3_O_4_) were evaluated at 310 K. All the nanoparticles possessed superparamagnetic behaviours, while the octahedral magnetic nanoparticles possessed much higher magnetization compared to the spherical ones (Fig. 2A). The O-Fe_3_O_4_-15 (−) nanoparticles had the highest saturation magnetization around 46.2 emu g^−1^, followed by S-Fe_3_O_4_-21 (−) nanoparticles of 32.1 emu g^−1^ and S-Fe_3_O_4_-10 (−) of 30.1 emu g^−1^. When zooming into the low magnetic field region (Fig. 2B), a hysteresis cycle in the O-Fe_3_O_4_-15 (−) group was observed and the coercive field was around 32 Oe. These six types of magnetic nanoparticles were used for evaluating the penetration in tumors, which comprised four variables in terms of their properties, including size, shape, surface charge and magnetization.

### 3.2. The cytotoxicity of Fe_3_O_4_ nanoparticles

The toxicity of the six types of Fe_3_O_4_ nanoparticles was assessed using MTT assay at the iron concentration ranging from 40 to 160 μg mL^−1^ for 24 h and 48 h (Fig. S3, ESI†). The results demonstrated that all positively surface charged Fe_3_O_4_ nanoparticles induced higher toxicity to MCF-7 cells than those with negative surface charges at all concentrations and time points. Within the three types of positively charged nanoparticles, at the highest concentration (160 μg mL^−1^) for 24 h or 48 h, the octahedral O-Fe_3_O_4_-15 (+) nanoparticles had the lowest toxicity. The three types of negatively surface charged nanoparticles induced very little toxicity for MCF-7 cells for 24 h or 48 h, at the highest concentration. Similarly, the negative octahedral 15 nm nanoparticles also displayed the lowest toxicity. The S-Fe_3_O_4_-10 (−) and S-Fe_3_O_4_-21 (−) nanoparticles showed similar toxicities at 160 μg mL^−1^ for 24 h (91% and 89% of cell viabilities, respectively), while the negative 10 nm nanoparticles displayed higher toxicity than negative 21 nm nanoparticles for 48 h (80% and 90% of cell viabilities at 160 μg mL^−1^ of Fe, respectively). These results suggested that spherical and positively surface charged nanoparticles are more toxic to MCF-7 cells.

### 3.3. Uptake of different Fe_3_O_4_ nanoparticles by monolayer cells

**Fig. 3:**
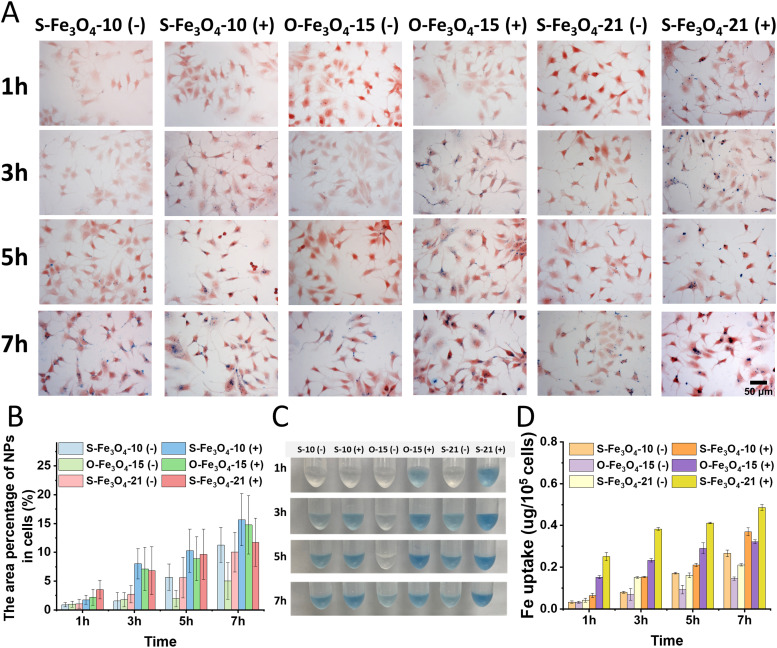
Cellular internalization of six types of Fe_3_O_4_ nanoparticles by MCF-7 monolayer cells and quantification of iron quantities. (A) Optical microscopy images of MCF-7 cells after 1, 3, 5, and 7 h of incubation with different Fe_3_O_4_ nanoparticles (20 μg Fe per mL) at 37 °C. The nanoparticles were stained with Prussian blue (blue) and cells were visualized by nuclei fast red staining (red). Scale bar, 50 μm. (B) Quantitative image analysis identifies the presence of Fe_3_O_4_ nanoparticle (NP) level in cells. The percentage area was averaged for individual cells (*n* = 30). (C) The digital images illustrating the Prussian blue colorimetric titration method to estimate the iron content in MCF-7 cells after 1, 3, 5, and 7 h of incubation with different Fe_3_O_4_ nanoparticles (60 μg Fe per mL) at 37 °C. (D) Quantification of Fe_3_O_4_ nanoparticle uptake by MCF-7 cells. The measurements were determined by Prussian blue titration (*n* = 3). All of the data are represented as the mean values ± standard deviation.

The cellular uptake of six types of Fe_3_O_4_ nanoparticles by MCF-7 cells was examined at different time points (1–7 h) and the nanoparticle amount associated with cells was determined by two methods, optical imaging and Prussian blue UV-vis titration. After 1 h of incubation, only the three types of positively charged nanoparticles were observed in the cytoplasm of cells (Fig. 3A and Fig. S4A, ESI†), which indicated that the positive surface charge can facilitate the rapid internalization of nanoparticles in cells. The three types of negatively charged nanoparticles started to appear in cells after 3 h of incubation, while the positively charged nanoparticles already displayed massive accumulation in cells at the same time point. The percentage of nanoparticles in individual cells was then quantitatively analyzed (Fig. S4B, ESI†). For all of the four studied time points, the three types of positively charged Fe_3_O_4_ nanoparticles displayed significantly higher iron accumulation than their negatively charged counterparts (Fig. 3B). Since the negatively charged nanoparticles maintained almost the same hydrodynamic sizes as their TEM sizes, we used the results from negatively charged nanoparticles to evaluate the impacts of the size and shape of nanoparticles on cellular uptake. At the end of the internalization, O-Fe_3_O_4_-15 (−) nanoparticles had the lowest blue area percentage in cells when compared with S-Fe_3_O_4_-10 (−) and S-Fe_3_O_4_-21 (−) nanoparticles. This suggests that the octahedral shape is not beneficial for cellular uptake. When we compared two different sizes of spherical nanoparticles, the iron areas of both nanoparticles increased with extended time point, the 21 nm nanoparticles had higher initial uptake efficiency before 3 h, but the 10 nm nanoparticles surpassed 21 nm nanoparticles at the endpoint (7 h) and had slightly higher area percentage. This explained why the 10 nm nanoparticles had higher cytotoxicity than the 21 nm ones at 48 h after incubation with cells.

To verify the cellular uptake results assessed by optical imaging, we further used the Prussian blue UV-vis titration method to test the internalization of different Fe_3_O_4_ nanoparticles in MCF-7 cells. The results showed similar trends to those from optical imaging observation. The positively charged nanoparticle groups showed a faster and stronger blue chromogenic reaction (Fig. 3C), which corresponded to higher cellular internalization than the negatively charged nanoparticles after quantitative analysis of the Fe content (Fig. 3D). The Fe content in cells for the three types of negatively charged nanoparticles still followed the trend that the O-Fe_3_O_4_-15 (−) nanoparticles were the lowest at all time points, the uptake of S-Fe_3_O_4_-21 (−) was the highest at the beginning while that of S-Fe_3_O_4_-10 (−) nanoparticles became the highest at the endpoint (7 h). Thus, these results confirmed that spherical and positively charged nanoparticles exhibit greater cellular internalization than octahedral and negatively charged nanoparticles. In addition, the results may suggest that within this small size range, the larger negative nanoparticles (21 nm) are capable of rapid cell internalization while the smaller negative nanoparticles (10 nm) have the advantage of high cellular accumulation after a relatively long incubation time.

### 3.4. The effects of size, shape and magnetic properties on the penetration of Fe_3_O_4_ nanoparticles in 3D tumor spheroids

**Fig. 4:**
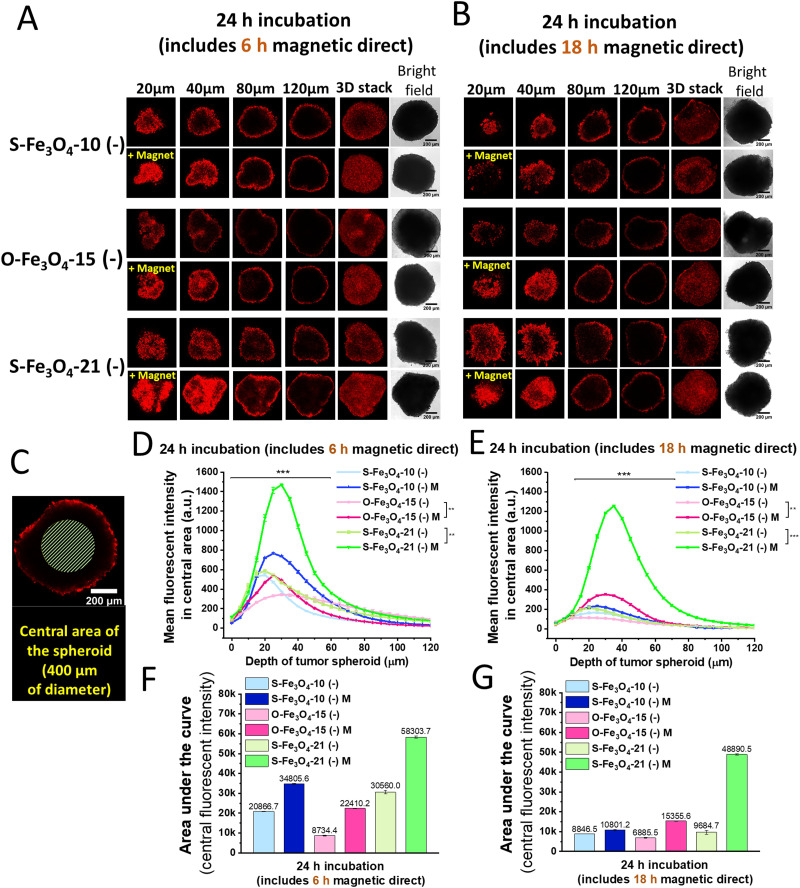
Tissue penetration of three types of negatively charged Fe_3_O_4_ nanoparticles (RBITC labelled) into 3D MCF-7 cancer cell spheroids for 24 h. (A and B) The fluorescence and bright field images of different transverse sections with depths from 20 to 120 μm of cell spheroids cultured for 24 h but with different magnetic control times, 6 h and 18 h. Control groups without applying a magnetic field. The groups with a magnetic field are labelled with ‘+Magnet’ on images. The 3D images were reconstructed using ImageJ by stacking transverse sections from 0 to 200 μm with a depth interval of 5 μm. The red color represents RBITC labelled Fe_3_O_4_ nanoparticles. Scale bar, 200 μm. (C) The schematic illustration of the central area selection of the transverse section (a circle with a diameter of 400 μm) to study the nanoparticle accumulation at different spheroid depths, which is to avoid counting nanoparticle signals on the spheroid surface. Scale bar, 200 μm. (D and E) The mean fluorescence intensity of Fe_3_O_4_ nanoparticles in the central area of transverse sections at different spheroid depths (*n* = 3). (F and G) The area under the curve calculated based on (D) and (E); the depths range from 0 to 120 μm (*n* = 3). (*P < 0.05, **P < 0.01, ***P < 0.001.) The error bars represent the standard deviation.

To investigate the effects of different properties of nanoparticles on the tissue penetration capacity, we used 3D tumor spheroids to mimic the human tumor structure.^36^ Because the surface charge had an influence on the loading efficiency of the fluorescence dye (Fig. S5, ESI†), we first checked the effects of size, shape and magnetic properties of Fe_3_O_4_ nanoparticles on the tumor spheroid penetration by labelling three types of negatively charged Fe_3_O_4_–DMSA nanoparticles (S-Fe_3_O_4_-10 (−), O-Fe_3_O_4_-15 (−), and S-Fe_3_O_4_-21 (−)) with RBITC. The tumor spheroids were incubated with nanoparticles under various conditions for total 24 h (18 h without + 6 h with magnetic application, 6 h without + 18 h with magnetic application, and 24 h without magnetic application. Afterwards, spheroids were imaged in every 5 μm section of tissue from the top to the middle using the Z-stack function in confocal laser scanning microscopy. For all of these nanoparticle groups, the spheroid incubated with nanoparticles upon magnetic field application (+magnet) displayed much stronger fluorescence of nanoparticles in 20–80 μm sections and stronger fluorescent signals were detected in the central area of spheroids in 3D stack images than the groups without magnetic field assistance (Fig. 4A and B). To compare the penetration and retention of different nanoparticles within the spheroids, we only quantified the fluorescence intensity in the central part of every section at different depths (a fixed circular area with a diameter of 400 μm) to remove the influence of signals from the nanoparticles that attached to the boundary of the spheroid (Fig. 4C). Compared with the groups without magnetic direction, application of magnetic field for 6 h or 18 h raised the intensities of all three nanoparticles at each section (depth interval is 5 μm) and moved the peak intensity positions to a deeper depth of spheroids (S-Fe_3_O_4_-10 (−) M, O-Fe_3_O_4_-15 (−) M, S-Fe_3_O_4_-21 (−) M) (Fig. 4D and E). Such results demonstrate that all three types of Fe_3_O_4_ nanoparticles can be driven by the magnetic force and penetrated deeper into tumor tissue. The area under the curve of every curve in Fig. 4D and E was then calculated, which reflects the total retention of nanoparticles in the central area of spheroids from the depth of 0 to 120 μm. Without the magnetic field application, the penetration and retention of the three types of nanoparticles in spheroids followed the following sequence: S-Fe_3_O_4_-21 (−) > S-Fe_3_O_4_-10 (−) > O-Fe_3_O_4_-15 (−) (Fig. 4F). The octahedral nanoparticles exhibited the lowest penetration capacity in tumor tissue, which corresponded to the result that the octahedral nanoparticles showed the lowest internalization in monolayer cells. When the penetration process was facilitated with 6 hours of magnetic field, the area under the curve for all three types of nanoparticles increased but still followed the following sequence of S-Fe_3_O_4_-21 (−) M > S-Fe_3_O_4_-10 (−) M > O-Fe_3_O_4_-15 (−) M. However, the increasing ratios of the area under the curve for the groups with a magnetic field to corresponding groups without magnetic field followed a different ranking order. O-Fe_3_O_4_-15 (−) M had the highest enhancement of penetration (2.56 times), higher than both S-Fe_3_O_4_-21 (−) M (1.91 times) and S-Fe_3_O_4_-10 (−) M (1.67 times). This ranking order is also the same as the order of magnetic properties of three nanoparticles that the octahedral 15 nm and spherical 10 nm nanoparticles exhibited the highest and the lowest magnetic strengths, respectively. Furthermore, when the initial incubation time point without magnetic field was reduced from 18 h to 6 h and then the magnetic field application time was increased from 6 h to 18 h (the total incubation time was kept constant for 24 h), the penetration and retention of O-Fe_3_O_4_-15 (−) M exceeded S-Fe_3_O_4_-10 (−) M although octahedral 15 nm nanoparticles still showed the lowest penetration without the magnetic field application (Fig. 4G). Besides, in 18 h magnetic field application groups, S-Fe_3_O_4_-21 (−) M still showed the best penetration efficacy in the spheroids. These results suggest that the cellular internalization and magnetic properties of Fe_3_O_4_ magnetic nanoparticles simultaneously affect their penetration in tumor tissue. The high magnetic reaction of nanoparticles to the external magnetic field can lead to improved penetration efficacy. While to obtain the highest penetration and retention in tumor, the initial cellular internalization of nanoparticles is also a crucial and determining factor. S-Fe_3_O_4_-10 (−) nanoparticles had good cellular internalization but weak magnetic properties. O-Fe_3_O_4_-15 (−) nanoparticles were the opposite, they had strong magnetic properties but very low cellular internalization. By contrast, S-Fe_3_O_4_-21 (−) nanoparticles exhibited good performance for both factors, and thus, they achieved the highest penetration and retention efficacy in tumor tissue.

### 3.5. The effect of surface charge on the penetration of Fe_3_O_4_ nanoparticles

**Fig. 5:**
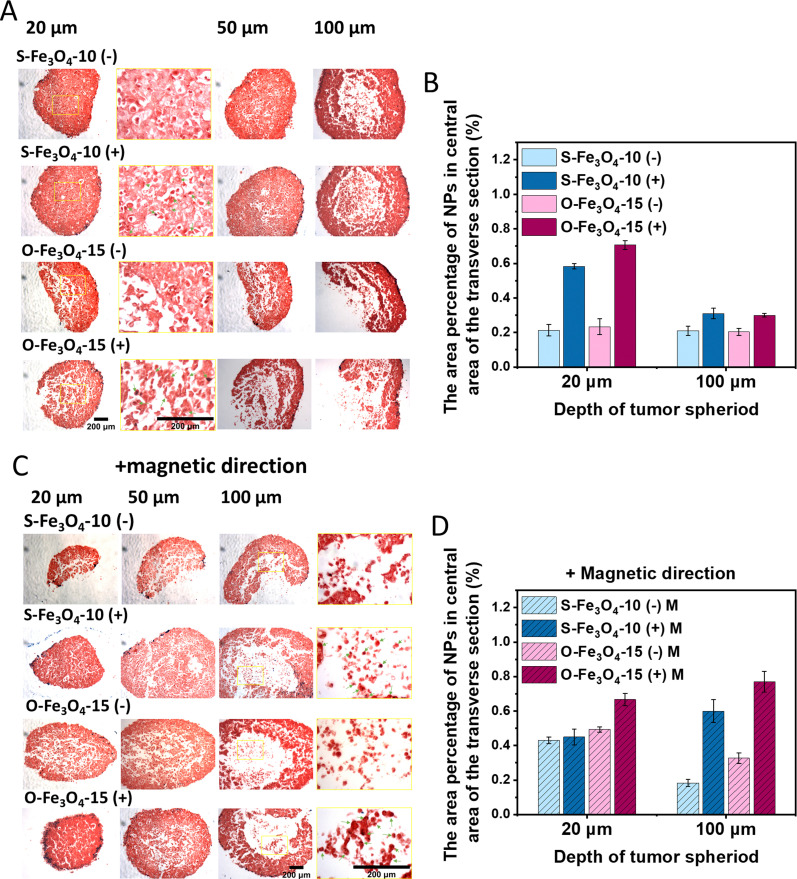
Penetration of negatively and positively surface charged Fe_3_O_4_ nanoparticles into MCF-7 cancer cell spheroids for 24 h. The representative optical microscopy images of tumor spheroid slices (depths at 20, 50 and 100 μm) which were incubated with Fe_3_O_4_ nanoparticles without a magnetic field as a control group (A) or with 16 h of magnetic field application (C). Fe_3_O_4_ nanoparticles were stained with Prussian blue (deep blue) and cells were stained with nuclear fast red (red). The green arrows in magnifying images indicate the uptake of Fe_3_O_4_ nanoparticles in the tumor center. Scale bar, 200 μm. (B and D) Quantitative analysis identifies the area percentage of Fe_3_O_4_ nanoparticles (NPs) in the central area of transverse sections (a circular area with a diameter of 600 μm) at spheroid depths of 20 and 100 μm when without (B) or with a magnetic field (*n* = 3) (D). The error bars represent standard deviation.

Next, we investigated the impact of the surface charge of nanoparticles on the tissue penetration capacity. Spherical 10 nm and octahedral 15 nm Fe_3_O_4_ nanoparticles with both negative and positive surface charges were tested in this section. For the condition without applying the magnetic field, after 24 h incubation, both the types of positively charged nanoparticles (S-Fe_3_O_4_-10 (+) and O-Fe_3_O_4_-15 (+)) displayed more attachment on the peripheral cell layer of spheroids than negatively charged ones (Fig. 5A). When the central area of a shallow depth of spheroids (20 μm) was zoomed in, more positively charged nanoparticles were observed in cells in this area while few negatively charged nanoparticles were found. The quantitative analysis further confirmed that the positive surface charge could significantly increase the ratio of Fe_3_O_4_ nanoparticles in a shallow area of spheroids (20 μm) and slightly increase the uptake of Fe_3_O_4_ nanoparticles in deep tissue areas (100 μm) (Fig. 5B). When the magnetic field was applied, the two types of positively charged nanoparticles were detected in the central area at a much deeper depth of spheroids (100 μm) (Fig. 5C) but only a few negatively charged nanoparticles were observed at this depth. The results indicated that the positive surface charge could facilitate the penetration of nanoparticles in tumor tissue. Furthermore, we concluded that the penetration capacity of nanoparticles in tumor tissue should be associated with their cellular internalization efficiency because we confirmed that positively charged nanoparticles also had higher cellular uptake in 2D cell culture than negatively charged nanoparticles, when no magnetic field was used, as discussed in the earlier section. It is also noticed that the area percentages of S-Fe_3_O_4_-10 and O-Fe_3_O_4_-15 nanoparticles in a deep area of tumor tissue (100 μm) were close to each other for both negatively and positively charged groups, when without the magnetic direction (Fig. 5B). In comparison, both O-Fe_3_O_4_-15 (−) and O-Fe_3_O_4_-15 (+) nanoparticles showed higher percentages at 100 μm depth of spheroids than the corresponding negatively and positively charged S-Fe_3_O_4_-10 nanoparticles, when the magnetic field was applied for 16 h (Fig. 5D). This finding supported the conclusion in the previous section that Fe_3_O_4_ nanoparticles with stronger magnetization and larger size are more advantageous in magnetically actuated tissue penetration.

## 4. Conclusions

In this study, we have synthesized ultrasmall superparamagnetic Fe_3_O_4_ nanoparticles with various physicochemical properties, including negatively or positively charged spherical 10 nm, octahedral 15 nm, and spherical 21 nm nanoparticles. The octahedral 15 nm particles possessed the highest magnetization, followed by spherical 21 nm and 10 nm particles. We first investigated their ability to be taken up into monolayer cancer cells without amagnetic field. The results demonstrated that the spherical and positively charged Fe_3_O_4_ nanoparticles exhibited much higher uptake in cells compared to their octahedral and negative counterparts. Next, we developed a 3D cancer cell spheroid model to mimic the tumor tissue for examining the penetration depth and retention of various Fe_3_O_4_ nanoparticles. Without an external magnetic field, the positive nanoparticles showed more accumulation in the shallow area of tumor spheroids than the negative nanoparticles. On comparing these three types of the negative nanoparticles, the octahedral 15 nm particles showed the lowest penetration efficiency, while the spherical 10 nm and 21 nm particles demonstrated better and similar penetration. In the presence of a magnetic field gradient, the histological staining of spheroids revealed that a larger fraction of the positively charged nanoparticles was internalized by central tumor cells which were at a considerable depth of spheroids, compared to their negatively charged counterparts. For the three types of the negatively charged nanoparticles, the penetration and retention of spherical 21 nm particles significantly enhanced and were the highest. The octahedral 15 nm particles showed the lowest penetration efficiency. When we increased the use of magnetic field time, the spherical 21 nm particles were still the best for penetration because they had both relatively high initial accumulation in tumor tissue and magnetization, and a large volume. But the penetration of octahedral 15 nm particles overpassed that of spherical 10 nm particles since they had much higher magnetization than the spherical 10 nm particles and were subjected to a greater Lorentz force from the applied magnetic field.

Therefore, our findings suggest that the initial penetration efficiency of ultrasmall Fe_3_O_4_ nanoparticles should be relevant to their cellular internalization ability, which is dependent on their shapes and surface charges. When considering the use of magnetic actuation for improving penetration, the effects of size/volume and magnetization of ultrasmall magnetic nanoparticles obviously play more dominant roles. However, to obtain the best magnetically actuated penetration, the cellular internalization and magnetic force on nanoparticles should be considered simultaneously. Our findings are useful to guide the design of physicochemical properties of superparamagnetic Fe_3_O_4_ nanoparticles to achieve optimal magnetically actuated locomotion and penetration in tumors for effective biomedical applications.

## Conflicts of interest

There are no conflicts to declare.

## Supplementary Material

TB-011-D2TB02630A-s001
