# Supplementary material for: Ultrasmall superparamagnetic iron oxide nanoparticles for enhanced tumor penetration
Source: J Mater Chem B. 2023 Mar 3;11(15):3422–33. doi: 10.1039/d2tb02630a (PMC10091360; doi:10.1039/d2tb02630a)
Supplement: TB-011-D2TB02630A-s001 [file TB-011-D2TB02630A-s001.pdf]

Electronic Supplementary Material (ESI) for Journal of Materials Chemistry B.
This journal is © The Royal Society of Chemistry 2023

Supporting information

Ultrasmall superparamagnetic iron oxide nanoparticles for enhanced tumor penetration

Xue Feng,[a] Yuxiang Xue,[a] Sevil Gonca,[a] Kunlang Ji,[b] Mei Zhang,[a] Francisco R. García-García,[c] Quan Li,[d] Yi Huang,[c] Konstantin V. Kamenev,[c,e] and Xianfeng Chen*[a]

*Corresponding authors

[a] School of Engineering, Institute for Bioengineering, University of Edinburgh, The King's Buildings, Edinburgh EH9 3JL (UK)

Email: Michael.Chen@ed.ac.uk

[b] School of Chemistry, Centre for Science at Extreme Conditions, University of Edinburgh, The King's Buildings, Edinburgh EH9 3FD (UK)

[c] School of Engineering, Institute for Materials and Processes, University of Edinburgh, The King's Buildings, Edinburgh EH9 3JL (UK)

[d] School of Engineering, Institute for Energy Systems, University of Edinburgh, The King's Buildings, Edinburgh EH9 3JL (UK)

[e] School of Engineering, Centre for Science at Extreme Conditions, University of Edinburgh, The King's Buildings, Edinburgh EH9 3FD (UK)

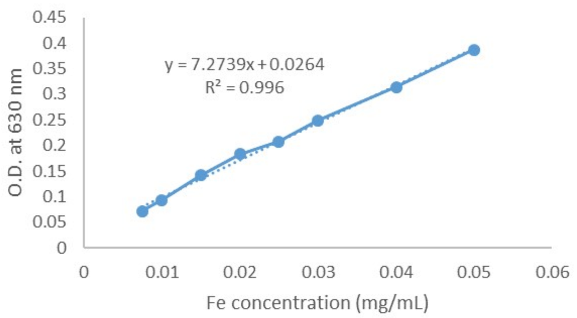

Figure S1. Standard curve of iron in 6M HCL via Prussian blue reaction.

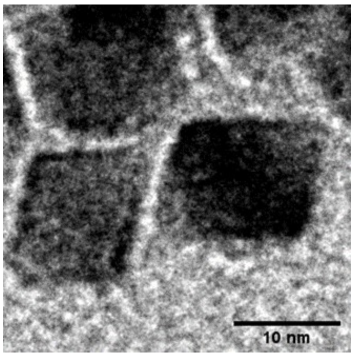

Figure S2. TEM image of typical octahedral 15 nm Fe_3O_4-OA nanoparticles. Scale bar, 10 nm.

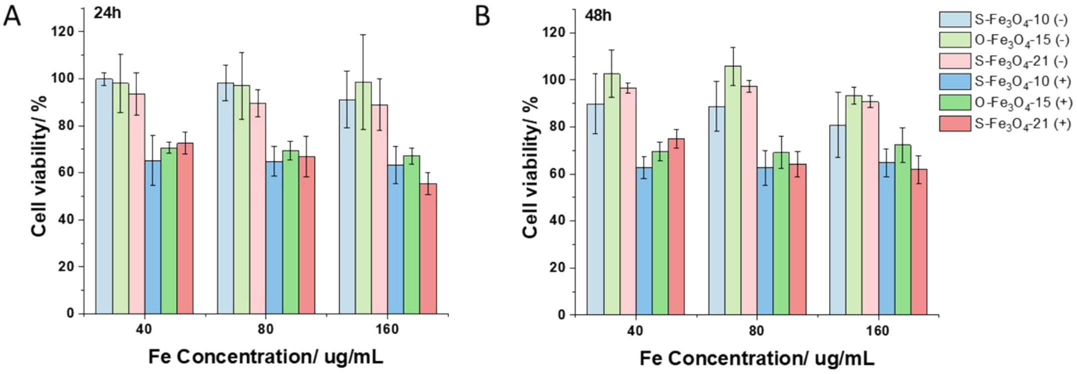

Figure S3. The cell viability of MCF-7 monolayer cells after treatment with different types of Fe_3O_4 nanoparticles at (**A**) 24 h and (**B**) 48 h. (Mean values ± standard deviation, n=5).

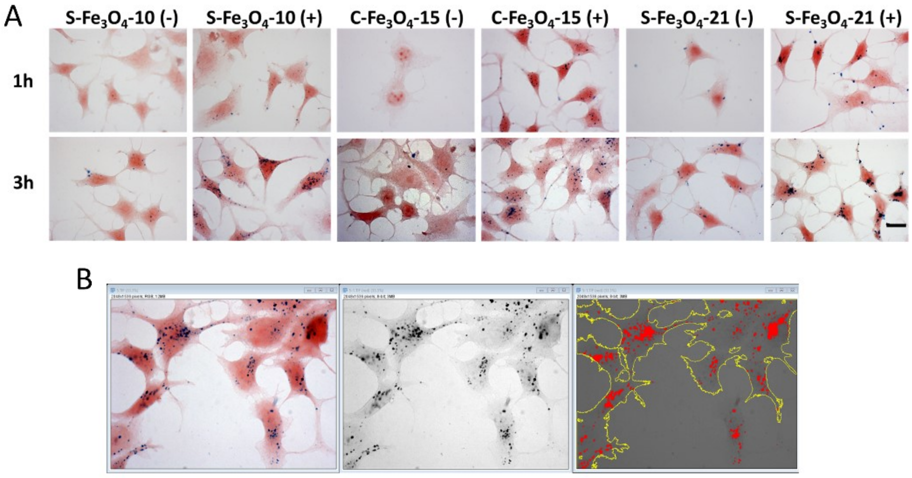

Figure S4. (**A**) Distribution of different types of nanoparticles in MCF-7 cells for incubation times of 1 h and 3 h. The cells were observed by an objective lens of 63X. Scale bar, 30 μm. (**B**) Pictures illustrated the process of calculating the relative area of the Fe_3O_4 nanoparticles to cells in ImageJ. The yellow lines indicate the cellular areas and the red dots indicate the accumulation of Fe_3O_4 nanoparticles.

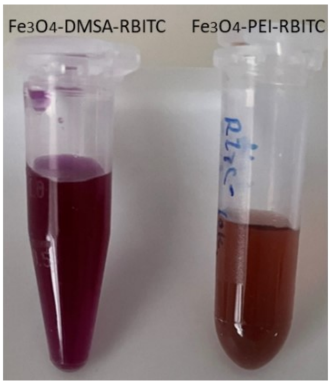

Figure S5. Picture displaying the Fe_3O_4-DMSA nanoparticles possessing higher dye loading efficiency than Fe_3O_4-PEI nanoparticles, showing deeper purple colour when the iron concentrations of the two types of nanoparticles are the same.

Table S1. Characterization of six types of Fe_3O_4 nanoparticles with different sizes, shapes and surface charges.

Sample name	Dynamic light scattering		Zeta potential (mV) (n=3)
	Size (Number mean, nm) (n=3)	Polydispersity index (PDI) (n=3)	
S-Fe_3O_4-10 (-)	12.9 ± 2.6	0.321 ± 0.02	-49 ± 2.3
O-Fe_3O_4-15 (-)	14.4 ± 0.2	0.42 ± 0.01	-30.8 ± 2.3
S-Fe_3O_4-21 (-)	23 ± 5.2	0.487 ± 0.007	-37 ± 1.4
S-Fe_3O_4-10 (+)	59.5 ± 1.7	0.209 ± 0.01	34.8 ± 0.6
O-Fe_3O_4-15 (+)	122 ± 14.8	0.179 ± 0.02	22.7 ± 2.6
S-Fe_3O_4-21 (+)	306.8 ± 8.1	0.594 ± 0.02	30.5 ± 0.2
